# A novel neuron-enriched protein SDIM1 is down regulated in Alzheimer's brains and attenuates cell death induced by DNAJB4 over-expression in neuro-progenitor cells

**DOI:** 10.1186/1750-1326-6-9

**Published:** 2011-01-21

**Authors:** Joy X Lei, Cristina G Cassone, Christian Luebbert, Qing Yan Liu

**Affiliations:** 1 Neurobiology Program, Institute for Biological Sciences, National Research Council of Canada, Ottawa, Ontario, K1A 0R6, Canada; 2 Faculty of Medicine, University of Ottawa, Ottawa, Canada

## Abstract

**Background:**

Molecular changes in multiple biological processes contribute to the development of chronic neurodegeneration such as late onset Alzheimer's disease (LOAD). To discover how these changes are reflected at the level of gene expression, we used a subtractive transcription-based amplification of mRNA procedure to identify novel genes that have altered expression levels in the brains of Alzheimer's disease (AD) patients. Among the genes altered in expression level in AD brains was a transcript encoding a novel protein, SDIM1, that contains 146 amino acids, including a typical signal peptide and two transmembrane domains. Here we examined its biochemical properties and putative roles in neuroprotection/neurodegeneration.

**Results:**

QRT-PCR analysis of additional AD and control post-mortem human brains showed that the SDIM1 transcript was indeed significantly down regulated in all AD brains. SDIM1 is more abundant in NT2 neurons than astrocytes and present throughout the cytoplasm and neural processes, but not in the nuclei. In NT2 neurons, it is highly responsive to stress conditions mimicking insults that may cause neurodegeneration in AD brains. For example, SDIM1 was significantly down regulated 2 h after oxygen-glucose deprivation (OGD), though had recovered 16 h later, and also appeared significantly up regulated compared to untreated NT2 neurons. Overexpression of SDIM1 in neuro-progenitor cells improved cells' ability to survive after injurious insults and its downregulation accelerated cell death induced by OGD. Yeast two-hybrid screening and co-immunoprecipitation approaches revealed, both *in vitro *and *in vivo*, an interaction between SDIM1 and DNAJB4, a heat shock protein hsp40 homolog, recently known as an enhancer of apoptosis that also interacts with the mu opioid receptor in human brain. Overexpression of DNAJB4 alone significantly reduced cell viability and SDIM1 co-overexpression was capable of attenuating the cell death caused DNAJB4, suggesting that the binding of SDIM1 to DNAJB4 might sequester DNAJB4, thus increasing cell viability.

**Conclusion:**

Taken together, we have identified a small membrane protein, which is down regulated in AD brains and neuronal cells exposed to injurious insults. Its ability to promote survival and its interaction with DNAJB4 suggest that it may play a very specific role in brain cell survival and/or receptor trafficking.

## Background

Alzheimer disease (AD) is the most common neurodegenerative disorder, manifesting clinical symptoms of cognitive impairment and dementia, which result from progressive synaptic dysfunction, loss and neuronal cell death. Pathologically, AD is characterized by the deposition of β-amyloid leading to the development of senile plaques and hyperphosphorylated tau protein aggregates within the cortical neurons, forming neurofibrillary tangles (NFTs). Our current understanding of early onset (familial) AD is derived primarily from studies on genes or gene products identified in genetically determined forms. These AD cases exhibit genetic linkage to mutations in presenilin-1 (PS1), presenilin-2 (PS2) and β-amyloid precursor protein (APP) genes [1]. Although these discoveries have been helpful in elucidating the basic molecular pathogenesis of familial AD, they only represent a relatively small fraction of the AD population. The large majority of cases are late onset AD (LOAD), which are genetically heterogeneous and occur sporadically. Several genetic risk factors have been described for LOAD, notably an allelic polymorphism of apolipoprotein E that affects the age of onset [2], but the precise etiology of LOAD is poorly understood.

Alterations in multiple biological processes contribute to the development of LOAD, some of which correlate with cognitive impairment [3]. Well established brain changes include excessive oxidative stress and insufficient antioxidant defenses, disrupted calcium homeostasis, altered cholesterol synthesis and transport, inappropriate hormonal and growth factor signaling, chronic inflammation, aberrant re-entry of neurons into the cell cycle and, especially, aberrant protein processing, folding and turnover, leading ultimately to senile plaques and NFT formation [4]. Due to the vast extent and complexity of these changes, global gene expression profiling has been adopted as a discovery-based approach to study this idiopathic and multifactorial disease. Although the discovery of concurrent changes in AD brains cannot establish cause and effect, or separate detrimental from compensatory effects, they can generate unique insights and testable hypotheses on processes that may drive brain and cognitive dysfunction.

The most commonly used technology for the assessment of gene expression changes in postmortem AD brains is DNA microarray analysis [5-9]. This approach has allowed relative quantitative assessment of thousands of genes simultaneously, providing clues for new candidate genes not previously associated with AD. However, this method requires prior knowledge of gene sequences and cannot be applied as a discovery tool for novel transcripts. Furthermore, the expression levels of low abundance genes cannot be readily assessed by DNA microarray hybridization, as reliable results are usually obtained only for genes that are expressed in high or moderate levels. We have recently employed a subtractive hybridization and RNA amplification method to enrich and isolate rare and novel transcripts involved in LOAD. Using this approach, we have isolated a number of novel transcripts that are differentially expressed in the brains of AD patients [10]. Among these was a novel sequence, SDIM1, that not only was down regulated in AD brains, but was also very responsive to stress conditions mimicking the injurious insults that may cause neurodegeneration in AD brain.

In the present study, we demonstrate its biochemical properties, tissue/cell type distribution, putative roles in neuronal cells that are exposed to toxic insults causing neurodegeneration, and have explored its relationship with other key proteins in the brain.

## Results

### SDIM1 is a novel transmembrane protein down regulated in AD brains

**Figure 1:**
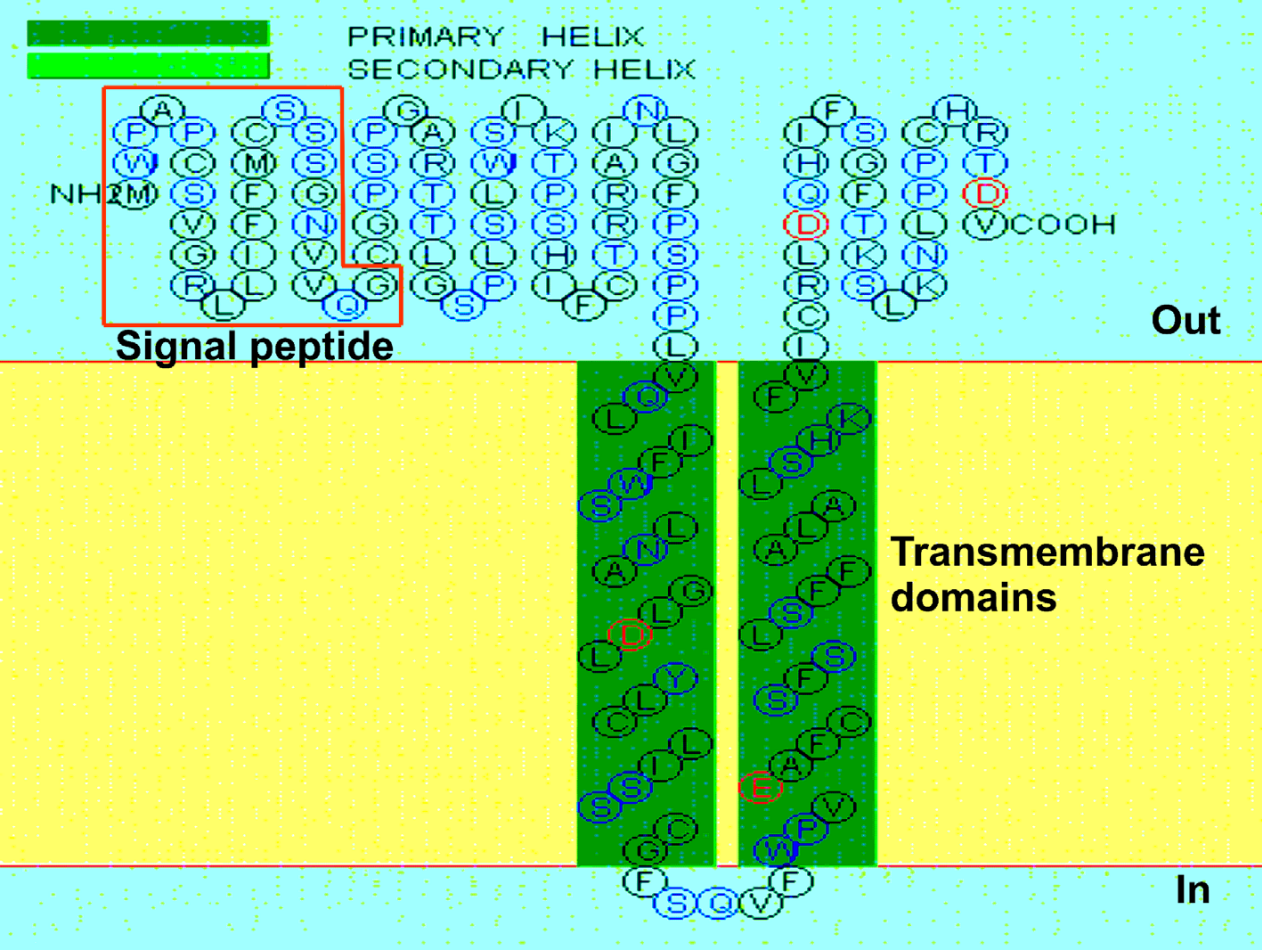
**A schematic representation of the human SDIM1 protein structures**. Structural motifs of the encoded protein were predicted by ExPASy tools (http://bp.nuap.nagoya-u.ac.jp/sosui/sosui_submit.html).

**Figure 2:**
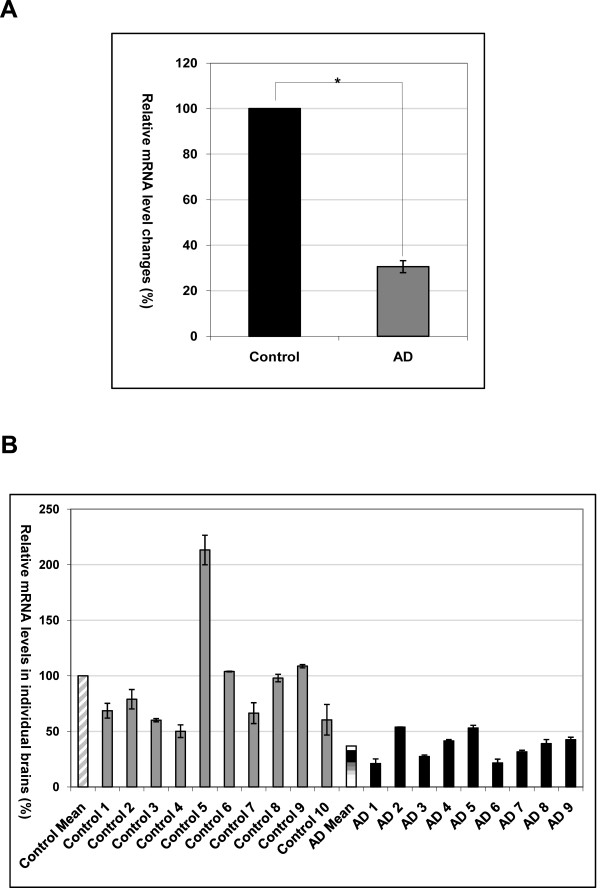
**Expression pattern of SDIM1 transcript in control and AD human brains**. **A**. Changes in mRNA levels of SDIM1 transcript in control and AD brains were determined by qRT-PCR. The cDNA samples were prepared from pooled mRNA of 4 AD and 5 age-matched control subjects. The value of the control sample was set at 100%. The percentage of the AD sample was calculated by 100x 2^-ΔCt^, where ΔCt is the cycle number difference between the AD sample and the control sample. The experiments were performed in triplicate. Asterisks indicate a significant difference (p = 0.0014) **B**. Changes in mRNA levels of SDIM1 transcript in individual control and AD brains were determined by qRT-PCR. The cDNA samples were prepared from mRNA of 9 AD and 10 age-matched control subjects. The qRT-PCR results were calculated against the average result (control mean) of the control samples, set at 100%. Percentage of each sample was calculated by 100 × 2^-ΔCt^, where ΔCt is the cycle number difference between each sample and the control mean. The experiments were performed in triplicate.

We have isolated a cDNA fragment by subtractive hybridization using a pooled mRNA population from AD brains as a "driver" and the first strand cDNAs from a pooled age-matched control brains as a "tester" [10]. BLAST searches revealed that this fragment matches with human cDNA FLJ26122 fis, clone SYN00634 on chromosome 6 [GenBank AK129633, AL590482], encoding an unnamed protein product [GenBank BAC85201.1] of 146 amino acids (aa), with a calculated molecular weight of ~13 kDa. The predicted primary structure of this protein contained a typical N-terminal signal peptide of 26 aa. There are two primary transmembrane helices spanning amino acids 69 to 91, 97 to 119, respectively (Figure 1). No matching cDNAs from rodents were found in the GenBank. Based on its structure and putative function determined by present study, we named this gene as stress responsive DNAJB4 interacting membrane protein 1 (SDIM1, HGNC approved). Since the SDIM1 transcript was found in the subtracted cDNA library containing genes potentially down regulated in AD brains, we re-examined these changes by qRT-PCR analysis (Figure 2A) of the RNA pools used to construct the original AD and control cDNA libraries [10]. These results were further confirmed by qRT-PCR using 19 individual brain samples from a tissue bank (Table 1). As shown in Figure 2B the SDIM1 transcript level was significantly lower in most AD brains in comparison to the age-matched controls, suggesting that SDIM1 transcript is indeed down regulated on AD brains.

**Table 1:** Description of brain samples used for qRT-PCR analysis of SDIM1

Patient	Sex	Age	Postmortem	Pathology
Control 1	Male	67	4.5 hours	Normal

Control 2	Female	88	4-8 hours	Normal

Control 3	Male	64	6 hours	Normal

Control 4	Female	N/A	4-8 hours	Normal

Control 5	Male	N/A	4-8 hours	Normal

Control 6	Male	89	9 hours	Normal

Control 7	Male	64	8 hours	Normal

Control 8	Male	63	8 hours	Normal

Control 9	Male	80	9.5 hours	Normal

Control 10	Male	95	11 hours	Normal

AD 1	Female	N/A	4-8 hours	AD, Dementia

AD 2	Male	N/A	4-8 hours	Moderate senile changes of AD type, dementia

AD 3	Male	N/A	6 hours	Subdural hematomas, senile changes of AD type

AD 4	Male	N/A	4-8 hours	Moderate senile changes of AD type, dementia

AD 5	Female	93	8 hours	Probable AD, according to CERAD, CVD cerebrum, right infarct old

AD 6	Female	84	8 hours	Probable AD, according to CERAD

AD 7	Female	85	7.5 hours	Probable AD, according to CERAD

AD 8	Male	77	6 hours	Senile dementia of AD type

AD 9	Female	86	7 hour	CERAD classification 2, definite AD

### Expression pattern of SDIM1

**Figure 3:**
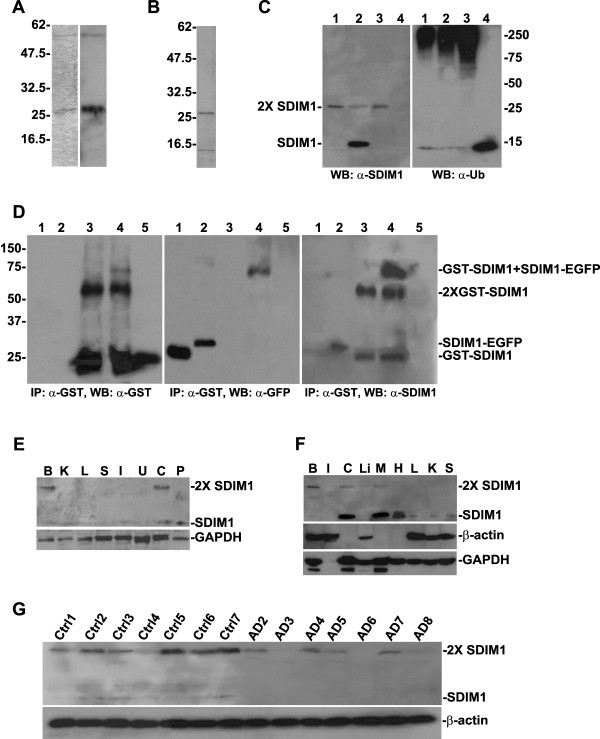
**Expression levels of SDIM1 protein in human and mouse tissues**. **A**. Anti-SDIM1 antibody was tested on recombinant GST-SDIM1 fusion protein (right lane). Left lane-Coomassie Blue stained GST-SDIM1. **B**. Anti-SDIM1 antibody detected SDIM1 in proteins extracted from NT2 neurons. **C**. SDIM1 protein from mouse brain (lane 1), muscle (lane 2) and HEK-293 cells over-expressing SDIM1 (lane 3) was detected with anti-SDIM1 (left panel) or anti-ubiquitin (right panel) antibody. Lane 4-purified human ubiquitin. **D**. GST-SDIM1 protein was mixed with proteins from HEK-293 cells transfected with pEGFP-N1 or pSDIM1-EGFP and immunoprecipitated with anti-GST antibody. The presence of monomeric and dimeric GST-SDIM1, GST-SDIM/SDIM1-EGFP or SDIM1-EGFP was detected by anti-GST (left panel) or anti-EGFP (middle panel) antibody. The second blot was stripped and probed with anti-SDIM1 anti-body (right panel). Lane1-total proteins from pEGFP-N1 transfected cells, lane 2-total proteins from pSDIM1-EGFP transfected cells, lane 3-mixture of GST-SDIM1 and protein from pEGFP-N1 transfected cells immunoprecipitated with anti-GST antibody, lane 4-mixture of GST-SDIM1 and protein from pSDIM1-EGFP transfected cells immunoprecipitated with anti-GST antibody, lane 5-mock immunoprecipitation without anti-GST antibody. **E**. The expression levels of SDIM1 protein in human tissues were detected by Western analysis with anti-SDIM1 antibody. A representative blot from each lot was tested by the vendor with anti-GAPDH antibody. Lane B-Brain, lane K-Kidney, lane L-Lung, lane S-Spleen, lane I-Small Intestine, lane U-Uterus, lane C-Cervix, lane P-Placenta. **F**. SDIM1 protein in mouse tissues was detected by Western analysis with anti-SDIM1 antibody. The striped membrane was re-blotted with anti-β-actin and anti-GAPDH antibodies. Lane B-Brain, lane I-Intestine, lane C-Cervix, lane Li-Liver, lane M-Muscle, lane H-Heart, lane L-Lung, lane K-Kidney, lane S-Spleen. **G**. SDIM1 protein in control and AD human brains were detected by Western analysis with anti-SDIM1 antibody.

**Figure 4:**
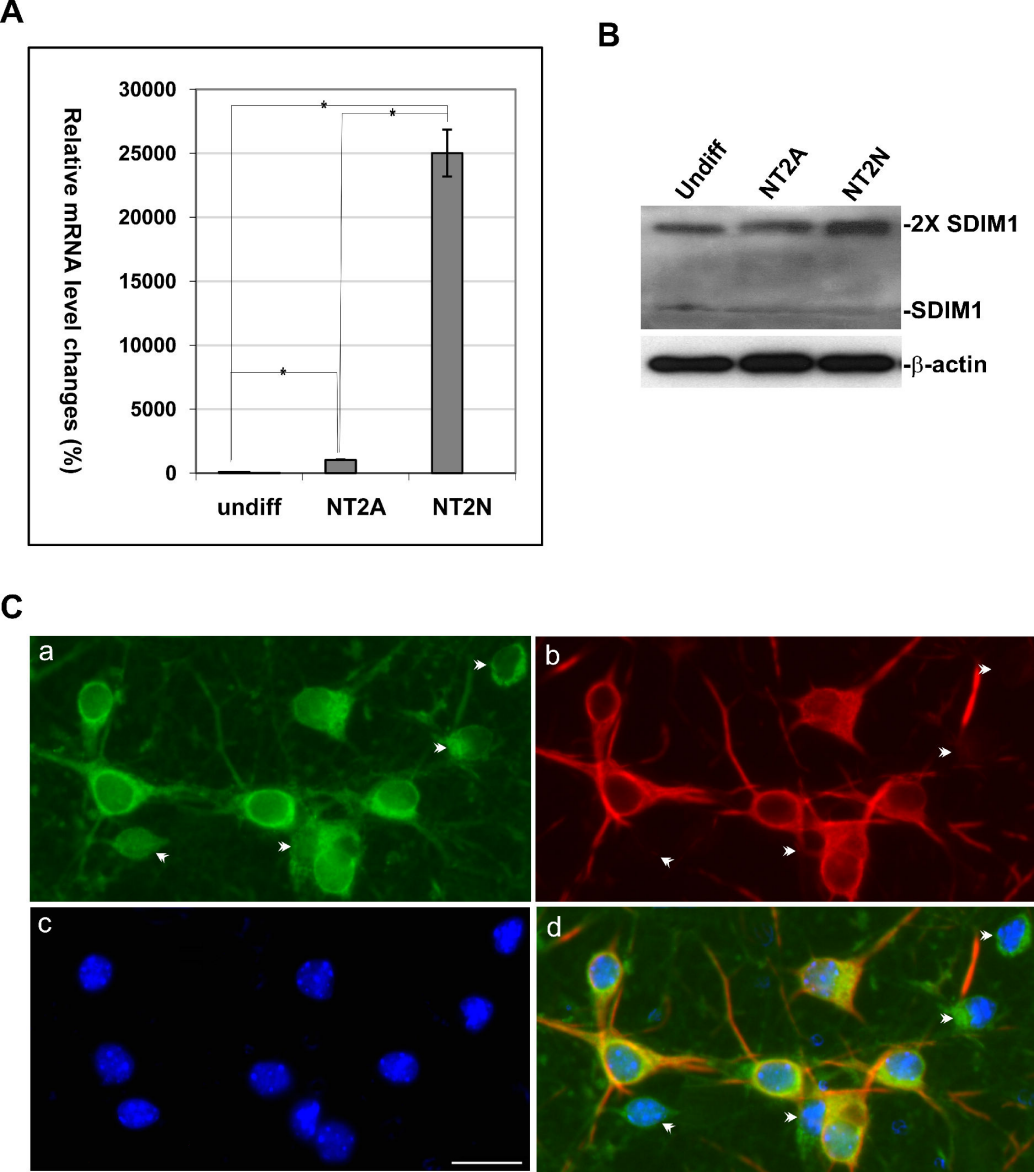
**Cell type specific expression and cellular localization of SDIM1**. **A**. Changes in mRNA levels of SDIM1 during RA-induced differentiation of NT2 cells were determined by qRT-PCR. The samples were measured against the cDNA of undifferentiated NT2 cells as a control, set at 100%. Percentage of each sample was calculated by 100 × p 2^-ΔCt^, where ΔCt is the cycle number difference between test sample and the control sample. undiff - undifferentiated NT2 cells (control), NT2A - NT2 astrocytes, NT2N - NT2 neurons. The experiments were performed in triplicate. Asterisks indicate a significant difference (p-value = 0.00024 for Undiff and NT2A; p-value = 0.0027 for Undiff and NT2N; p-value = 0.0029 for NT2A and NT2N). **B**. Changes in SDIM1 protein levels were determined by Western blotting with anti-SDIM1 antibody using 100 μg/lane of total cellular protein. The Western blotting of β-actin was shown as loading control. **C**. Subcellular localization of SDIM1. Mouse primary cortical neurons were fixed and stained with anti-SDIM1 and anti-MAP2 antibodies. Staining with secondary anti-body alone was also performed as a negative control (image not show). FITC-conjugated anti-rabbit IgG (for SDIM1) and rhodomine-conjugated anti-mouse IgG (for MAP2) were used to detect the specific immunostaining. The nuclei were stained with DAPI and viewed with a Zeiss Axiovert 200 M fluorescence microscope. Arrowheads indicate glial cells or inmature neurons. Scale bar in c = 50 μM.

Since the SDIM1 sequence exists in the GenBank as a hypothetical protein, we set out to produce a specific anti-SDIM1 antibody that can detect SDIM1 gene product in tissues and cell lines. A polyclonal anti-SDIM1 peptide antiserum was generated in rabbits and further purified by immumoaffinity methods. This anti-SDIM1 antibody faithfully recognized recombinant GST-SDIM1 fusion protein (Figure 3A, right lane) and detected two bands from the protein extract from NT2 neurons (Figure 3B). The lower band was located near 13-15 kDa and the upper one appears at 26-27 kDa on SDS-PAGE, which suggests that SDIM1 might form dimeric aggregates under denaturing conditions. Modifying the concentrations of DTT, β-mercaptoethanol, and urea in the loading buffer, as well as changing the boiling temperature from 100°C for 5 Min to 55°C for 30 min did not break up these aggregations. SDIM1 protein sequence contains two potential phosphorylation sites and two O-linked glycosylation sites. There is no N-linked glycosylation site on this protein. The size increase resulted from these modifications do not seem to match the size shift seen on the gel (Figure 3B). Mono-ubiquitination may be a possibility for the upper band that is recognized by anti-SDIM1 antibody. However, neither band was recognized by anti-ubiquitin antibody in western analysis experiments (Figure 3C). Throughout the present study, we noticed that the intensity of the upper band was always more pronounced than the lower band, the upper band increased as the lower band decreased and the lower band could disappear completely if the same protein extracts had been freeze-dried or been passed through multiple freeze-thaw cycles prior to gel separation. This phenomenon led us to believe that the band shift was a result of dimeric aggregation, instead of enzymatic modification, which normally occurs under physiological conditions *in vivo*. To prove that SDIM1 forms dimeric structure, we performed co-immunoprecipitation experiments, where cellular proteins from cells transfected with pEGFP-N1 or pSDIM1-EGFP plasmids were mixed with purified GST-SDIM1 fusion protein. Immunoprecipitation was performed using anti-GST antibody and the precipitated proteins were detected by anti-GST, anti-EGFP and anti-SDIM1 antibodies. These experiments showed that GST-SDIM1 formed dimmers (Figure 3D, left panel, lane 4, also see Figure 3A) and it also formed hetero-dimers with SDIM1-EGFP (Figure 3D, left panel, lane 4 and middle panel, lane 4). SDIM1-EGFP also formed self-dimmers as we observed a faint band in the lane with total cellular proteins extracted from pSDIM1-EGFP transfected cells (Figure 3D middle panel, lane 2). The dimeric structures on the membrane shown in figure 3D, middle panel, were further detected by anti-SDIM1 antibody (Figure 3D right panel) and we did not detect dimerization of EGFP with GST-SDIM1 (Figure 3D middle and right panels, lane 3). Western analyses revealed ubiquitous expression of SDIM1 in human and mouse tissues with higher levels in brain, muscle, heart and reproductive organs (Figure 3E and 3F). SDIM1 dimer was the dominant form in the brain, where the monomer was almost non-detectable. We also observed small amounts of dimers in freshly extracted cervix and muscle samples, where SDIM1 is more abundant (Figure 3E and 3F). Western analysis of proteins extracted from control and AD brain context showed that the overall SDIM1 protein levels in AD samples were indeed lower than those of controls (Figure 3G). We only observed trace amounts of SDIM1 monomer in control samples, but it was not detectable in the AD samples. Quantitative RT-PCR analysis detected very low levels of SDIM1 transcript in undifferentiated NT2 cells and NT2 derived astrocytes, but with significantly increased expression in NT2 neurons (Figure 4A). Its abundance in neurons was further confirmed by Western analysis using anti-SDIM1 antibody (Figure 4B). In these cells the dimer was also seen as the dominant form of SDIM1. To examine the cellular localization of SDIM1, we double stained mouse primary neuronal cultures with SDIM1 and MAP2 antibodies. SDIM1 protein is located mainly in the cytoplasm and distributed throughout the neuro-processes (Figure 4C a). Although SDIM1 has similar cellular distribution as that of MAP2 in mature neurons, it is not co-localized with MAP2 in the processes (Figure 4C d). Consistent with the Western analysis results shown in 4B, in the immature neurons, where there was no MAP2 immunoreactivity, we observed less SDIM1 staining than in mature neurons (Figure 4C arrowheads), suggesting that SDIM1 is more abundant in neuronal cells in the brain.

### SDIM1 is highly responsive to stress conditions mimicking injurious insults

**Figure 5:**
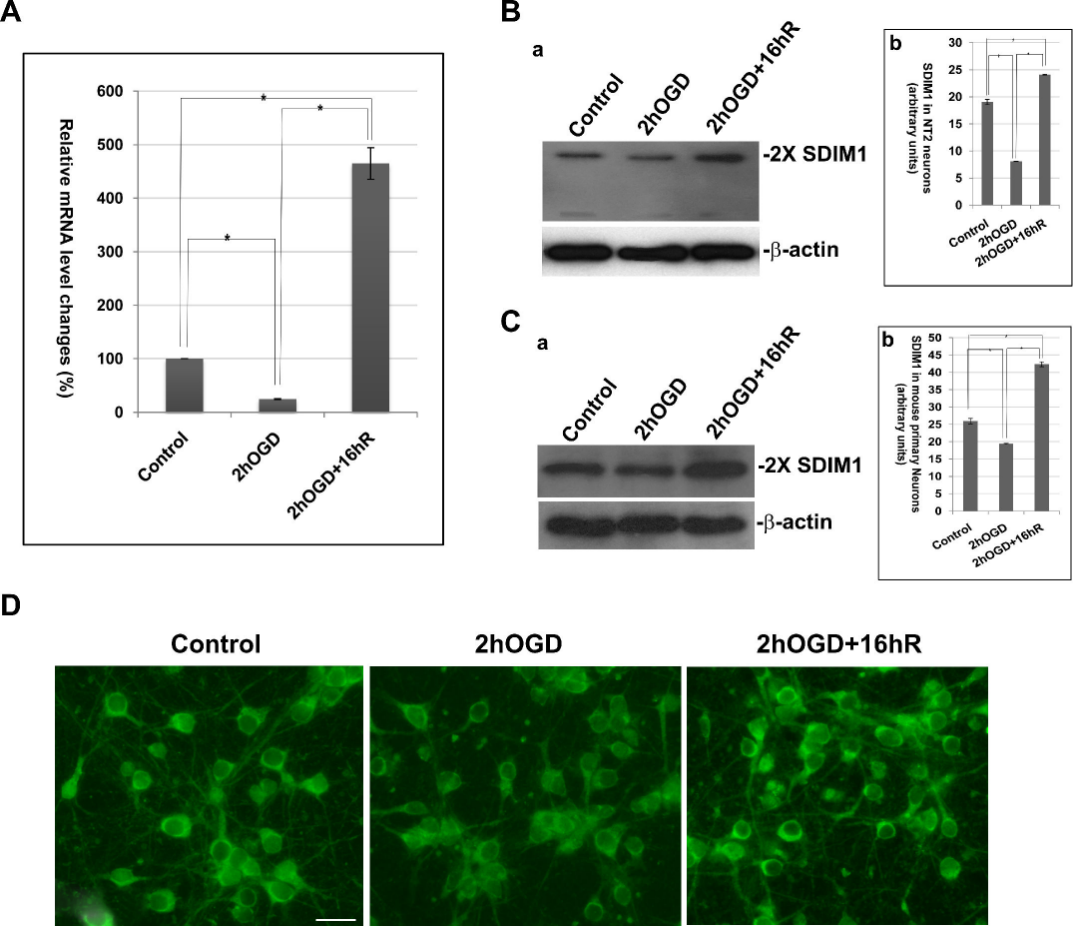
**Bi-phasic response of SDIM1 in NT2 and mouse primary neurons treated with cell death-inducing stress**. **A**. Changes in mRNA levels of SDIM1 in NT2 neurons treated with OGD and OGD with 16 h recovery were determined by qRT-PCR. The samples were measured against the cDNA of untreated NT2 neurons, set at 100%. Percentage of each treated sample was calculated by 100 × 2^-ΔCt^, where ΔCt is the cycle number difference between treated sample and the control sample. The experiments were performed in triplicate. Asterisks indicate a significant difference (p-value = 6.25E-05 for control and 2hOGD; p-value = 0.0032 for control and 2hOGD + 16hR; p-value = 0.0023 for 2hOGD and 2hOGD + 16hR). **B**. **a**. Changes in SDIM1 protein levels in NT2 neurons treated with OGD and OGD plus 16 h recovery were determined by Western blotting with anti-SDIM1 antibody. Band intensity was measured using ImageJ and the level of SDIM1 was normalized against the level of β-actin and plotted in **b**. Asterisk indicates a significant difference (ρ < 0.005; t-test). **C**. **a**. Changes in SDIM1 protein levels in mouse primary neurons treated with OGD and OGD plus 16 h recovery were determined by Western blotting with anti-SDIM1 antibody. Band intensity was measured using ImageJ and the level of SDIM1 was normalized against the level of β-actin and plotted in **b**. Asterisk indicates a significant difference (ρ < 0.005; t-test). **D**. Changes of immunoreactivity of SDIM1 in primary neurons treated with OGD and OGD plus 16 h recovery. Cells were stained with anti-SDIM1 antibody. Cy3-conjugated anti-rabbit IgG was used to detect the specific immunostaining. The stained cells were viewed with a Zeiss Axiovert 200 M fluorescence microscope. Scale bar = 50 μM.

We analyzed the SDIM1 expression level in post-mitotic NT2 neurons subjected to oxygen and glucose deprivation (OGD), which has been previously reported to trigger neuronal apoptosis [11]. Here, initially the cells were subjected to 2 h OGD treatment, during which 10-15% of cells lost viability, followed by a 16 h recovery period, at the end of which cell death reached 35-40%. The SDIM1 mRNA was significantly down regulated after the OGD treatment (Figure 5A), but became significantly up regulated 16 h after recovery. The changes in protein level, as verified by Western blot analysis, followed the same pattern (Figure 5B). Cultured mouse primary neurons responded to OGD treatment in the same fashion as that of the NT2 neurons (Figure 5C). Immunostaining of these OGD treated primary neurons showed that they had decreased immunoreactivity to anti-SDIM1 antibody and the cells appeared to have retracted their processes (Figure 5D). However, 16 h after re-oxygenation, the surviving cells had much brighter SDIM1 staining and normal neural processes. Dead or dying cells contained much less SDIM1 protein (data not shown). Taken together, our results indicated that SDIM1 had a bi-phasic response to cell death inducing injuries - a down regulation in response to the initial treatment, followed by significant up regulation in surviving cells.

### Overexpression of SDIM1 protected cells from injurious insults and its downregulation accelerated cell death caused by OGD in neuronal progenitor cells

**Figure 6:**
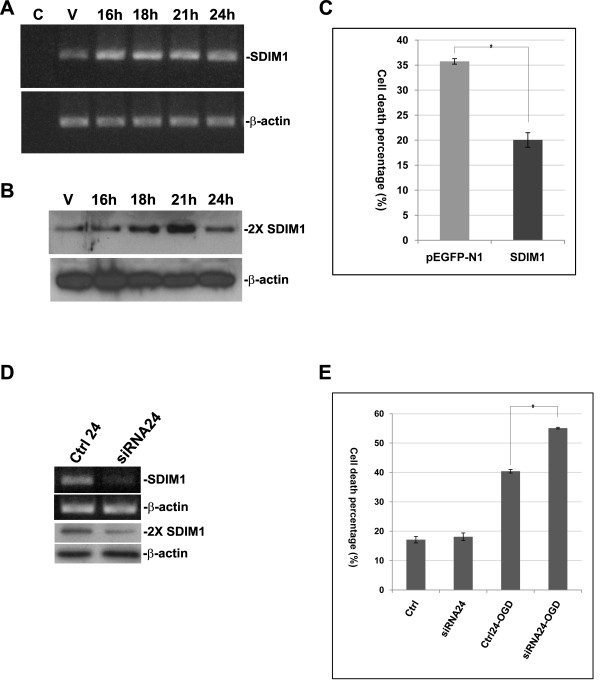
**Cellular levels of SDIM1 modulate the rates of cell death**. N2a or ERK293 cells were transiently transfected with pSDIM1*EGFP plasmid. NT2 cells were transfected with synthetic siRNAs. Cells were collected for total RNA and protein extractions 16-24 h after transfections. Trypan Blue exclusion assay was performed 21 or 24 h after transfection or 16 h after a 6 h OGD treatment of the transfected samples. Over expression of SDIM1 mRNA was assessed by RT-PCR (**A**) and Western analysis (**B**). Lane C - negative PCR control, lane V- mock transfection with pEGFP-N1 vector, lane 16 h - transfection with pSDIM1*EGFP plasmid for 16 h, lane 18 h - transfection with pSDIM1*EGFP plasmid for 18 h, lane 21 h - transfection with pSDIM1*EGFP plasmid for 21 h, lane 24 h - transfection with pSDIM1*EGFP plasmid for 24 h. **C**. Percentage of cell death after pSDIM1*EGFP transfection for 21 h. Bars represent the percentage of cell death in the population (mean ± SEM from 3 independent experiments performed in duplicate). Asterisk indicates a significant difference (p-value = 0.0096). **D**. Assessment of the siRNA silencing efficiency. RNA and protein samples were collected 24 after transfection with siRNAs. Down regulation of SDIM1 was analyzed by RT-PCR and Western blotting. **E**. Percentage of cell death 24 h after siRNA transfection, with or without OGD treatment. Bars represent the percentage of cell death in the population (mean ± SEM from 3 independent experiments performed in duplicate). Asterisks indicate a significant difference (p-value = 0.001). Ctrl24 - 24 h after transfection with negative control siRNAs, siRNA24 - 24 h after transfection with SDIM1 pool siRNAs, Ctrl24-OGD - 24 h after transfection with negative control siRNAs plus OGD treatment, siRNA24-OGD - 24 h after transfection with pool SDIM1 siRNAs plus OGD treatment.

To better understand the role of SDIM1 in neuroprotection we examined the effects of gene over-expression in the N2a cell line. We cloned the coding region of SDIM1 into a mammalian expression vector, pEGFP-N1, with a stop codon inserted between the end of the SDIM1 and the beginning of EGFP. As a result, a faint green fluorescent signal was observed in the cells transfected with the plasmid construct, but the SDIM1 protein was free to perform its routine function without the possible interference of the EGFP. A time course experiment was performed in order to determine a time point, when SDIM1 protein reached the plateau. While there was a gradual increase in protein level from 16 h to 21 h after transfection, we were surprised to discover that SDIM1 protein level deceased quickly after it reached the plateau at 21 h. Although the mRNA level remained much higher than the untransfected control, protein levels dropped to the same level 24 h after transfection (Figure 6A and 6B). This result suggests that there is a degradation mechanism controlling the protein level of SDIM1. Therefore over-expression of SDIM1 for 21 h was chosen for subsequent over-expression and OGD treatment experiments. When SDIM1 was over expressed in N2a cells without any cell death inducer, we did not observe any changes in cell viability. However, when the transfected cells were challenged by OGD treatment, we observed a significant decrease in cell death when compared with cell populations transfected with pEGFP-N1 vector control (Figure 6C). Next, we down regulated the endogenous SDIM1 in NT2 cells using a mixture of four siRNAs targeting human SDIM1. Most of the SDIM1 transcript and protein were knocked down 24 h after the siRNA transfection (Figure 6D). The transfected cells (after 24 h) were subjected to 6 h OGD treatment plus 16 h re-oxygenation and examined for cell viability (Figure 6E). When endogenous SDIM1 was knocked down by siRNA alone, no significant increase in cell death percentage was observed. However, down-regulation of the endogenous SDIM1 followed by OGD treatment significantly increased the percentage of cell death caused by OGD treatment.

### SDIM1 interacts with a DnaJ-like heat shock protein

**Figure 7:**
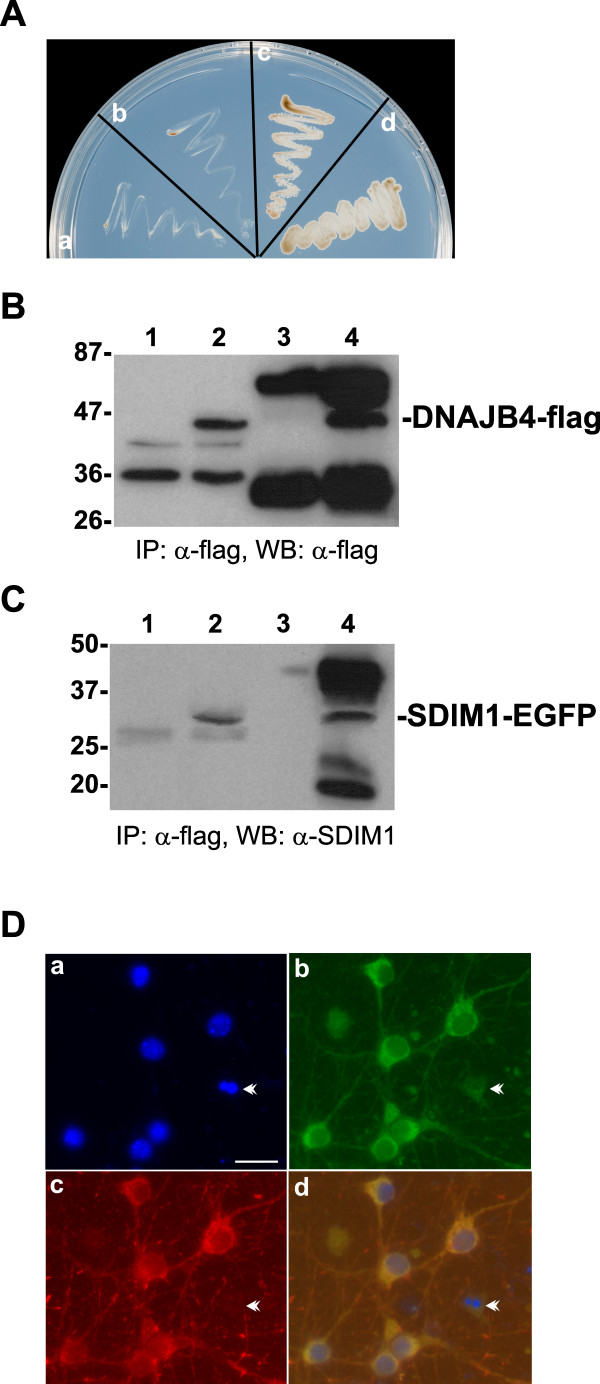
**SDIM1 physically interacts with DNAJB4**. **A**. Interaction between SDIM1 and DNAJB4 in yeast two-hybrid system. Empty or SDIM1 containing pGBKT7 bait vector and empty or DNAJB4 containing pACT2 library vector were co-transformed into yeast host cells AH109 and plated onto SD/-Trp -Leu -Ade -His + X-α-gal plate. **a **- a negative test of empty bait vector and DNAJB4, **b **- a negative test of SDIM1 bait plus empty library vector, **c **- a positive test showing the interaction between SDIM1 and DNAJB4, **d **- a standard positive control showing interaction between p53 and T antigen. **B**. Total cellular proteins were extracted from HEK-293 cells co-transfected with flag-tagged DNAJB4 and EGFP-tagged SDIM1 constructs and immunoprecipitated with anti-flag antibody. The presence of DNAJB4 in the complex was detected by anti-flag antibody. Lane1 - total cellular proteins extracted from untransfected cells, lane 2 - total cellular proteins extracted from cells co-transfected with pCMV-DNAJB4-Tag1 and pSDIM1-EGFP, lane 3 - mock immunoprecipitation without primary anti-flag antibody, lane 4 - proteins immunoprecipitated with anti-flag antibody. **C**. The presence of SDIM1 in the complex, shown in **B**, was revealed by Western blotting with anti-SDIM1 antibody. Lane assignment is the same as those in **B**. **D**. Co-localization of SDIM1 and DNAJB4. Mouse primary neurons were fixed and stained with anti-SDIM1 anti-DNAJB4 antibodies. FITC-conjugated anti-rabbit IgG (for SDIM1) and rhodomine-conjugated anti-mouse IgG (DNAJB4) were used to detect the specific immunostaining. The nuclei were stained with DAPI and viewed with a Zeiss Axiovert 200 M fluorescence microscope. **a**. DAPI stained nuclei. **b**. Anti-SDIM1 staining. **c**. Anti-DNAJB4 staining. **d**. **a**, **b **and **c **overlay. Arrowheads indicate an apoptotic cell. Scale bar in a = 50 μM.

To further identify the roles of SDIM1 in neuronal cell death or survival we performed yeast two-hybrid screening to identify its potential interacting proteins in human brain. Yeast strain AH109 harboring the two-hybrid construct (pGBKT7-SDIM1) expressing full length human SDIM1 was used to screen a human brain expression cDNA library. Among 4 clones that displayed Ade/His prototype and β-galactosidase activity, one sequence was found to encode human DnaJ-like heat shock protein (DNAJB4), also known as HLJ1. The interaction between these two proteins was reproducibly reconstructed in the yeast two-hybrid system and it passed all required tests (Figure 7A). Because the endogenous levels of SDIM1 and DNAJB4 were very low in most cell lines, in order to establish the interaction of these two proteins *in vivo*, we co-transfected flag-tagged DNAJB4 and EGFP tagged SDIM1 plasmids into HEK 293 cells. The transfected cells were harvested and the protein extracts were immmunoprecipitated with anti-flag antibody. The precipitated protein complex was boiled and separated by SDS/PAGE. The blot was first probed with anti-flag antibody to ensure that DNAJB4 protein was successfully precipitated by the anti-flag antibody (Figure 7B). The same protein samples were subsequently probed with anti-SDIM1 or anti-EGFP antibody for SDIM1 (Figure 7C). Our results confirmed the interaction detected through the yeast two-hybrid assay. Next, we examined the sub-cellular localization of these proteins by double staining of mouse primary neurons with anti SDIM1 and DNAJB4 antibodies. Co-localization of these proteins was detected in the cytoplasm and neuroprocesses (Figure 7D). Decreased amounts of SDIM1 were detected in apoptotic cells; only trace amounts of DNAJB4 was detected in these dying cells (Figure 7D, b and 7c, arrowhead). This result is in agreement with the physical interaction detected in the yeast two-hybrid and co-immunoprecipitation assays.

### SDIM1 attenuates cell death induced by DNAJB4 overexpression

**Figure 8:**
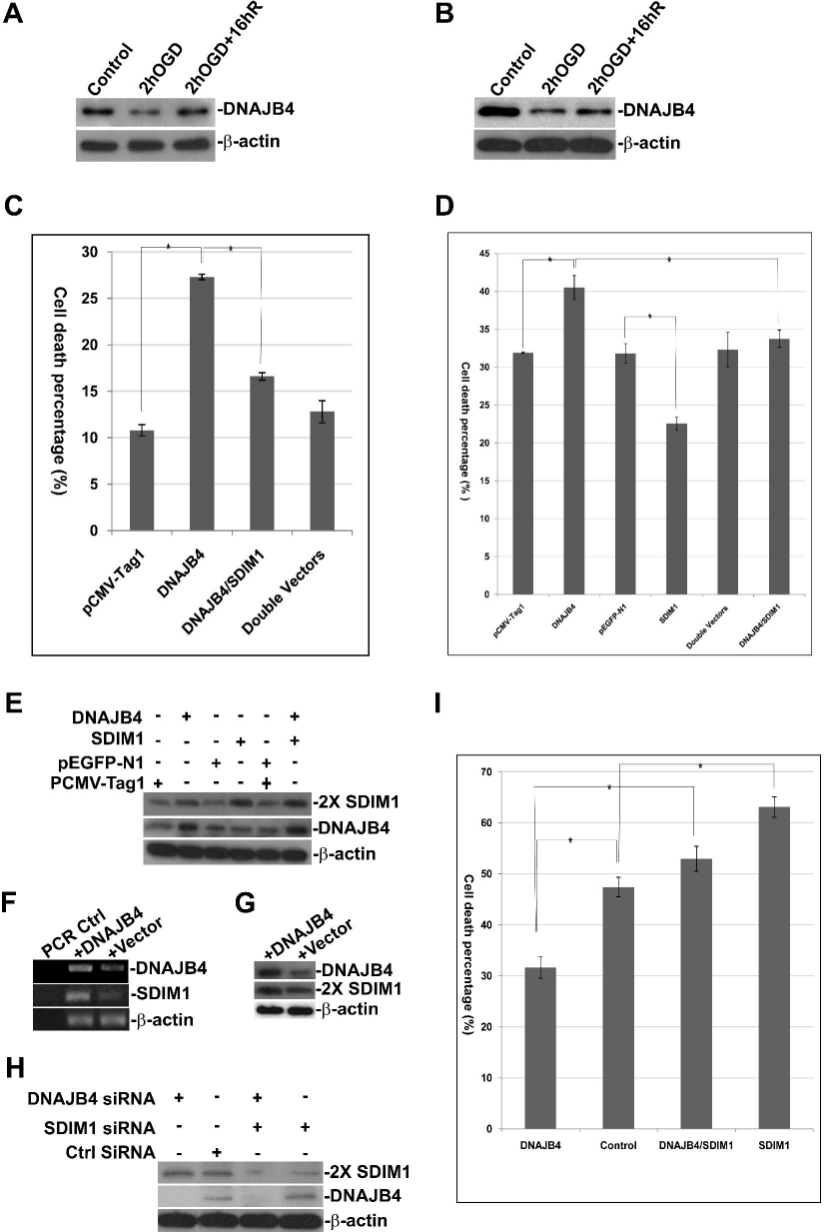
**SDIM1 attenuates cell death induced by DNAJB4 overexpression**. Changes in DNAJB4 protein levels in NT2 neurons (**A**) and mouse primary neurons (**B**) treated with OGD and OGD plus 16 h recovery were determined by Western blotting with anti-DNAJB4 antibody. **C**. Percentage of cell death after DNAJB4 overexpression alone or co-overexpression with SDIM1 in N2a cells. Cell death percentage was assessed 21 h after transfection. Bars represent the percentage of cell death in the population (mean ± SEM from 3 independent experiments performed in duplicate). Asterisk indicates a significant difference (p-value = 0.0016 for pCMV-Tag1 and DNAJB4; p-value = 0.0022 for DNAJB4 and DNAJB4/SDIM1). **D**. Percentage of cell death of the transfected cells after OGD treatment. The single or double transfected cells shown in **C **were treated with OGD for 6 h and allowed to recover for 16 h. The cell death assay was performed as described for **C**. (p-value = 0.0033 for pCMV-Tag1 and DNAJB4; p-value = 0.0027 for pEGFP-N1 and SDIM1; p-value = 0.057 for DNAJB4 and DNAJB4/SDIM1). **E**. Assessment of gene overexpression efficiency. Protein samples were collected 21 h after transfection. Overexpression of SDIM1 and DNAJB4 proteins was analyzed by Western blotting. **F**. RT-PCR analysis of RNA levels of DNAJB4 and SDIM1 in N2a cells transfected with pCMV-DNAJB4 or control vector pCMV-tag1. **G**. Western analysis of protein levels of the same samples shown in **F**. **H**. Assessment of siRNA silencing efficiency. NT2 cells were transfected or double transfected with synthetic siRNAs targeted for DNAJB4 and/or SDIM1. Cells were collected for protein extractions 24 h after transfection. **I**. Percentage of cell death 24 h after siRNA transfection followed by OGD treatment. (p-value = 0.032 for DNAJB4 and control; p-value = 0.029 for DNAJB4 and DNAJB4/SDIM1; p-value = 0.023 for control and SDIM1).

To understand the biological significance of the interaction between SDIM1 and DNAJB4, we first examined the expression patterns of DNAJB4 in AD brains and in cultured NT2 and primary neurons. We did not detected significant changes of DNAJB4 transcript in AD brains by qRT-PCR (data not show). We detected a bi-phasic change of DNAJB4 protein in NT2 and mouse primary neurons after OGD treatment and re-oxygenation similar to that observed with SDIM1 (Figure. 8A and 8B). However, we did not observe an increased expression of DNAJB4 in surviving cells in these neuronal cultures 16 h after re-oxygenation. The DNAJB4 level in the recovering cells (after re-oxygenation) was always lower than that of untreated cells. These data suggested that DNAJB4 may not have a protective function similar to SDIM1 in surviving cells. To test this hypothesis, we transiently over-expressed DNAJB4 in N2a cells with a pCMV-DNAJB4-Tag1 plasmid for 21 h. Surprisingly, over-expression of DNAJB4 triggered significant amounts of cell death as compared with mock transfection of empty vector, and co-transfection of DNAJB4 with SDIM1 attenuated cell death caused by DNAJNB4 over-expression (Figure 8C). The subsequent challenge of these transfected cells with OGD caused additional loss of cells in all samples, but the prior observations were reproduced in that SDIM1 over-expression reduced cell death caused by both OGD treatment and DNAJB4 over-expression (Figure 8D). The over-expression of DNAJB4 and SDIM1 proteins in the transfected cells were confirmed by Western analyses (Figure 8E). To investigate whether DNAJB4 induced cell death is through SDIM1 degradation mediated by DNAJB4; we performed a time course experiment of DNAJB4 over-expression and assessed the levels of endogenous SDIM1. While we detected a consistent increase of DNAJB4 levels at 16, 18, 21 and 24 hour time points due to the transfection of pCMV-DNAJB4-tag1 plasmid, we did not observe a decrease of SDIM1 protein at any time point (data not show); instead, we found a significant increased of SDIM mRNA and protein levels 24 h after DNAJB4 over-expression (Figure 8F and 8G). Next, we down regulated the endogenous DNAJB4 alone or SDIM1 and DNAJB4 simultaneously using synthetic siRNAs targeting human DNAJB4 and/or SDIM1 in human NT2 cells. The proteins were knocked down 24 h after siRNA transfection (Figure 8H). The transfected cells were then subjected to 6 h OGD treatment and 16 h re-oxygenation in a normal culture chamber and examined for cell viability (Figure 8I). The results showed that down regulation of DNAJB4 significantly reduced the percentage of cell death caused by OGD treatment. In contrast, down regulation of SDIM1 significantly increased cell death percentage and when both proteins were knocked down the cell death percentage returned to levels similar to control cells (Figurs. 6E, 8I), suggesting that endogenous DNAJB4 can promote cell death triggered by OGD and endogenous SDIM1 promotes cell survival.

## Discussion

We have isolated a novel neuron-enriched protein, SDIM1, which is down regulated in AD brain tissues. Further study of its expression in NT2 cell model and in mouse primary neurons revealed that this gene was barely detectable in undifferentiated NT2 cells, and is much more abundant in differentiated neurons than astrocytes. Treatments of neuronal cells with OGD and OGD plus 16 h recovery triggered a bi-phasic response of SDIM1 transcript and protein. SDIM1 is down regulated 2 h after OGD, but became highly up-regulated 16 h after re-oxygenation, in surviving neurons. Furthermore, over-expression of SDIM1 in N2a cells protected the cells from apoptosis caused by OGD insults. Conversely, down-regulation of SDIM1 by siRNA alone did not trigger cell death, whereas down-regulation of SDIM1 followed by OGD treatment of the cells caused accelerated cell death. These results suggest that SDIM1 is capable of promoting cell survival and this protective function is induced by signals triggered by stress conditions.

Although analysis of human and mouse tissues indicated that SDIM1 is relatively abundant in muscle, heart and reproductive organs, suggesting that this protein may have a basic housekeeping function under normal physiological conditions, it is the down regulation in AD brains that links SDIM1 to diseased conditions. SDIM1 transcript level is significantly lower in the cortex of AD patients than that of age matched controls. In our cell systems treated with injurious insults, SDIM1 is also down regulated and only becomes up regulated in surviving cells when given a recovery period. These results indicate that the surrounding microenvironment of the AD brain presents a constant stressed condition for brain cells. Neuroinflammation, oxidative stress, ischemia and Aβ deposition could all contribute to the creation of this harmful environment. Based on the siRNA knock down experiments, down regulation of SDIM1 alone is not enough to kill cells. However, when downregulation of SDIM1 in the cells combined with injurious insults caused increased amounts of cell death, suggesting that a decreased level of SDIM1 in the microenvironment of AD brains may likely cause neuronal cell death.

Through yeast two hybrid and co-immunoprecipitation assays we have discovered that SDIM1 interacts with DNAJB4, a member of DNAJ like heat shock protein 40. DNAJ/HSP40 proteins have been characterized as co-chaperones involved in the regulation of HSP70 chaperone activities [12]. DNAJB4 has been recently identified as a novel tumor suppressor of non-small-cell lung cancer (NSCLC) that can inhibit cancer cell cycle progression, proliferation, invasion and tumorigenesis. DNAJB4 expression was lower in tumors than adjacent normal tissues and patients with high DNAJB4 expression tumors had reduced cancer recurrence and longer survival than those with lower expressing tumors [13]. Studies on its regulatory mechanism revealed that DNAJB4 is transcriptionally up regulated via enhancer activator protein-1 (AP-1) binding to promoter Yin Yang-1 (YY1) and the co-activator, p300 [14,15]. Further investigations on the mechanism of action of DNAJB4 on tumor progression, particularly on its role in apoptosis of lung cancer cells exposed to UV stress, has indicated that DNAJB4 promotes UV induced apoptosis through JNK and caspase-3 activation in NSCLC cell line. DNAJB4 is a substrate of caspase-3 and this cleavage of DNAJB4 by caspase-3 is required for UV induced apoptosis in CL1-5 cells stably overexpressing DNAJB4. Consequently, DNAJB4 is reduced in UV exposed cells, and almost completely depleted by the late stage of apoptosis [16]. The results on the role of DNAJB4 in apoptosis obtained in the present study is, in principle, consistent with those obtained from NSCLC cell lines, in that over-expression of DNAJB4 in N2a cells promotes apoptosis. Silencing DNAJB4 decreases apoptosis induced by OGD (Figurs. 8C, 8I,). The difference is that transient over-expression of DNAJB4 alone in N2a cells can trigger apoptosis without any apoptotic inducer. This discrepancy is likely due to the fact that the NSCLC cell over expressing DNAJB4 is a cell line that had passed through a selection procedure for stable, forced-expression of exogenous DNAJB4. Many cells that initially over-express DNAJB4 would undergo apoptosis and might not survive long enough to form stably transfected clones. Further studies are required to identify whether the underlying molecular mechanism of DNAJB4 triggered cell death in neuro-progenitor cells is similar to that in NSCLC cells.

DNAJB4 is identified in the present study as a protein that interacts with a novel neuron-enriched protein SDIM1 that is down regulated in AD brains. However, we did not detect significant changes of DNAJB4 in AD brains. The fact that DNAJB4 protein is quickly depleted in apoptotic neurons and in NSCLC makes it difficult to quantitate whether there exists an initial up regulation of DNAJB4 which triggers the cascade of neurodegeneration in AD brains. The decreased amounts of DNAJB4 in cultured NT2 or primary neurons either during OGD treatment or after the recovery period is also likely due to the depletion of this protein in apoptotic cells. In these cell models, SDIM1 is highly responsive to OGD treatment. It's up regulation in the surviving cell population is highly pronounced, implying a protective function. Over-expression of DNAJB4 triggered an increase of endogenous SDIM1 suggesting that DNAJB4 induced cell death is not due to SDIM1 degradation mediated by DNAJB4; instead, this over-expression can trigger SDIM1 up-regulation, thus protect cells from DNAJB4 induced apoptosis (Figure 8F and 8G). This protective function appears to be independent of OGD treatment, because over-expression of SDIM1 attenuated apoptosis caused by DNAJB4 over-expression without any OGD treatment (Figure 8C). On the other hand, knocking down SDIM1 by siRNA silencing increases cell death induced by OGD (Figure 6E), indicating that SDIM1 also protects cells from apoptosis caused by OGD. Taken together, our data suggest that SDIM1 is a novel protein that possesses protective function against stress induced cell death.

It has been reported that DNAJB4 interacts with the carboxyl tail of human mu opioid receptor in the cell membrane [17]. While the functional relevance of this interaction still remains elusive, since DNAJB4 interacts with HSP70, and the latter is involved in synaptic transmission and regulation, it was speculated that DNAJB4 might work in concert with other HSP proteins to play a role in receptor trafficking.

## Conclusions

We have isolated a novel neuron-enriched protein, SDIM1, which is down regulated in AD brains. Further study of its function in neuronal cell models indicates that this protein is protective of neuro-progenitor cells from OGD induced cell death. Its physical interaction with DNAJB4, which is an apoptotic enhancer in NSCLC cells, and its ability to attenuate DNAJB4 mediated apoptosis suggest an anti-apoptotic function of SDIM1. However, further studies are necessary to understand the role of DNAJB4 in brain tissues, especially in the context of AD, and the protective mechanism of SDIM1 under stressed conditions in human brain.

## Methods

### Cell culture and oxygen-glucose deprivation (OGD) treatment

Human embryonal teratocarcinoma Atera2/D1 (NT2) cells (Stratagene, La Jolla, CA), mouse Neuro-2a (N2a) neuroblastoma cells (ATCC CCL-131) and human HEK 293 cells [18] were cultured in Dulbecco's modified Eagle's medium (Invitrogen, Bethesda, MD) supplemented with 10% fetal calf serum (GCS, Wisent, Inc. St. Bruno, PQ). NT2 cells were differentiated into neurons and astrocytes with all trans-retinoic acid (RA, Sigma, Oakville, ON) according to the method of Pleasure and Lee [19] as described previously [20]. Mouse primary cortical neurons were prepared from embryonic E15-16 CD1 mice and cultured in neurobasal media for 5-14 days as previously described [21].

For OGD treatment, NT2 neurons and mouse primary neurons were washed once with glucose-free DMEM, and incubated in glucose-free DMEM with 10% FBS for 2 h in a hypoxic chamber (Forma 1025 Anaerobic Chamber; ThermoForma, Marietta, OH, USA). At the end of the OGD treatment, cells were removed from the chamber and returned to the incubator for 16 h. The same OGD treatment was performed with undifferentiated NT2 and N2a cells except the incubation in OGD conditions was increased to 6 h due to their resistance to OGD treatment. Cell viability for all cell lines was assessed by the Trypan Blue (Sigma, Oakville, ON) exclusion assay. In this procedure, all floating cells were collected from culture media and washing buffer, and then combined with the trypsinized cells. Cells were incubated in the Trypan Blue dye for 5 min. Labelled cells were counted using a hemocytometer.

### RNA extraction, real time quantitative RT-PCR (qRT-PCR) and semi-quantitative RT-RCR

RNA extraction, first strand cDNA synthesis, and qRT-PCR analysis were performed as described previously [22]. RNA pools extracted from frontal cortex of postmortem human brain samples described previously [23] were used for subtractive hybridization and qRT-PCR. Additional brain samples were obtained from the Human Brain and Spinal Fluid Resource Center ([24], VAMC, Los Angeles, CA), which is sponsored by NINDS/NIMN, National Multiple Sclerosis Society, VA Greater Los Angeles Healthcare System, and Veterans Health Services and Research Administration, Department of Veteran Affairs. To detect the expression level of the SDIM1 transcript in brain tissue and cultured cells, equal amounts of cDNA (2 ng each) were used with the primers: SDIM1F 5' GGGCCATGAACACATCACTTG 3' and SDIM1R 5' TCAGGTCAAAGTTGGCAATGAA 3'. The DNAJB4 transcript was detect by using the primers: DNAJB4F 5' TCTCAAACAAGACCCTCCCA 3' and DNAJB4R 5' ATGGCAGCCCATATCCAATA 3'. PCR was performed using a ABI 7500 FAST Real Time PCR system and reagents according to the manufacturer's instructions. The primers used for semi-quantitative RT-PCR for SDIM1 are: F 5' TCGGTGGGAAGACTGCTTAT 3' and R 5' CGTCTGTCCTGTGACATGGT 3'; for DNAJB4 are: F 5' TGGTTGTACCAAACGGATGA 3' and R 5' ATGGCAGCCCATATCCAATA 3'.

### Plasmids, transient transfections and staining

Human cDNA encoding the mature (without the signal peptide) SDIM1 protein was cloned into the pGEX -3X vector (GE Healthcare, Baie d'Urfe Quebec) for GST-SDIM1 fusion protein production. The first amino acid after the signal peptide (position 27) was mutated from "C" to "A" in order to increase the solubility of the recombinant SDIM1 protein in E. coli. Human cDNA encoding full length SDIM1 protein was cloned in the pEGFP-N1 vector (Clontech, Palo Alto, CA, USA) with or without a stop codon added between the C-terminus of SDIM1 and the EGFP sequence (Invitrogen, Burlington, ON) to produce pSDIM1*EGFP, pSDIM1-EGFP respectively. The coding region of the human DNAJB4 cDNA was cloned into the pCMV-Tag1 vector to create a pCMV-DNAJB4-Tag1 construct.

For overexpression analysis, N2a cells were plated in 6-well plates at a density of 0.5 × 10^6 ^cells/well, 24 h before transfection. Cells were transfected with 5 μg pCMV-DNAJB4-Tag1 plasmid or pSDIM1*EGFP plasmid and 15 μl lipofectAmine 2000 reagent, or co-transfected with 2.5 μg each of the pSDIM1*EGFP and pCMV-DNAJB4-Tag1 plasmids plus 15 μl lipofectAmine 2000 reagent. Cells were collected for Trypan Blue exclusion assay as well as total RNA and protein extraction 16-24 h after transfection; or treated with OGD for 6 h plus 16 h recovery prior to Trypan blue assay or total RNA and protein extraction. For siRNA silencing, the siRNAs were purchased from GenePgarma (Shanghai GenePgarma Co, Ltd, Shanghai, PRC). Undifferentiated NT2 cells were plated in 12-well plates at a density of 0.25 × 10^6 ^cells/well, 24 h before transfection. Cell were transfected with 100 μM mixed human SDIM1 siRNAs containing a pool of two sequences: 5' UUAAACAGAGAUAUAAGUC 3' and 5' UUUAAUAGACCACAAACUC 3' and/or 100 μM mixed human DNAJB4 siRNAs containing three sequences: 5' UUGGAUAGUCUAGCACUUC 3', 5' UUUCUUCAGAAUCUCUACC 3' and 5' UUUCGAGAAAUCUUCAUCC 3' using Dharmafect1 transfection reagent according the manufacturer's instructions (Dharmacon, Thermo Fisher Scientific, Inc). Cells were subjected to 6 h OGD treatment 24 h after transfection and collected for Trypan Blue exclusion assay 16 h after re-oxygenation. For co-immunoprecipitation analysis, HEK 293 cells were plated in 10 cm plates at a density of 2 × 10^6 ^cells /plate, 24 h before transfection. Cells were transfected with 15 μg of pEGFPN1 or pSDIM1-EGFP alone, or co-transfected with 7.5 μg each of pSDIM1-EGFP and pCMV-DNAJB4-Tag1 plasmids DNA mixed with 45 μl LipofectAmine 2000 reagent. Cells were collected for total protein extraction 21 h after transfection. For cellular localization of SDIM1, mouse primary cortical neurons were stained (or double-stained) with anti-SDIM1 (dilution1:100 v/v), anti-MAP2 (1:200 v/v dilution, Novus Biologicals, Inc Littleton, CO), or anti-DNAJB4 (1:100 v/v dilution, abcam, Cambridge, MA) antibodies, followed by FITC -conjugated anti-rabbit IgG, rhodomine-conjugated anti-mouse IgG (for MAP2 and DNAJB4). The nuclei were counterstained with DAPI in PBS for 5 min and then mounted in Vectashield mounting medium (Vector laboratories, Burlingame CA, USA). The cells were viewed with a Zeiss Axiovert 200 M fluorescence microscope equipped with a Zeiss AxioCam camera (Zeiss, Midland, ON). The images were captured and analyzed using Zeiss Axiovision 3.1 software.

### Antibody production and purification

Custom polyclonal antibody (GenScprit, Piscataway, NJ) was produced using synthetic peptide N'-LGSPLSLWSIKTPS. The immune serum was purified by immunoaffinity purification using recombinant GST-SDIM1 fusion protein. Briefly, purified GST-SDIM1 fusion protein was separated by SDS-PAGE and electro-blotted onto a nitrocellulose membrane. The Ponceau stained membrane portion containing the SDIM1 antigen was excised and subjected to a Western blotting procedure using 2 mL original crude serum. The bound antigen-specific antibody was eluted with 0.1 M Glycine-HCl buffer, pH 2.7. The eluted antibody was neutralized by adding 1/10 volume of 1 M Tris, pH 8.5, concentrated using Amicon Ultra-15 Centrifugal Filter Device (Millipore, Fisher Scientific, Ottawa, ON).

### Protein extraction, Western blotting and co-immunoprecipitation

Recombinant GST-SDIM1 fusion protein was purified from Rosetta cells using a glutathione column according to the manufacturer's instruction (GE Healthcare, Baie d'Urfe Quebec). Mouse tissues were frozen in liquid nitrogen and homogenized in RIPA buffer in an electronic homogenizer and then kept on ice for 45 min. The samples were centrifuged at 14,000 xg for 10 min at 4°C to collect the supernatant for total cellular proteins. For total protein extraction from cultured cells, cells were trypsinized and collected by centrifugation. They were washed twice with PBS and lysed with RIPA buffer containing 1X protease Inhibitor cocktail (Roche Diagnostics, Indianapolis, IN). The lysate was vortexed and incubated on ice for 15 min, followed by sonication for 30 sec. In some cases the total cellular protein was freeze-dried and reconstituted in PBS in order to achieve a higher concentration. Western blotting analyses were performed as previously described [25]. Human tissue protein blot was purchased from BioChain Institute, Inc (Hayward, CA, USA). Each lane contains 50 μg of total cellular protein. The blots were probed with the following primary antibodies: Rabbit polyclonal, affinity-purified anti-SDIM1 (1:5000), rabbit polyclonal anti-flag (1:1000, Rockland, Gilbertsville, PA), mouse monoclonal anti-DNAJB4 (1:1000, Abcam, Cambridge, MA), mouse monoclonal anti-EGFP (1:1000, Milipore, Temecula, CA), goat polyclonal anti-GST (1:1000, Amersham Phamacia Biotech, Baie d'Urfe, QC), mouse monoclonal anti-ubiquitin (1:1000) and mouse monoclonal anti-β-actin (1:5000, both Sigma, Oakville, ON), and mouse monoclonal anti-GAPDH (1:10,000 v/v, Milipore, Temecula, CA). The antigens were detected using horseradish peroxidase-conjugated secondary antibodies: anti-mouse IgG (1:5000 v/v), anti-rabbit IgG (1:5000 v/v, both from Jackson ImmunoResearch Laboratories, Inc., West Grove, PA) or anti-goat IgG (1:5000, Sigma, Oakville, ON). The antigen-antibody complexes were visualized by enhanced chemiluminescence using an ECL Plus detection kit (Amersham Phamacia Biotech, Baie d'Urfe, QC).

For the co-immmunoprecipitation assay, flag-tagged DNAJB4 and EGFP-tagged SDIM1 constructs were transiently co-transfected into HEK-293 cells and total cellular proteins were extracted as described above. In another case, purified GST-SDIM1 protein was mixed with cellular proteins extracted from HEK-293 cells transfected with pEGFP-N1 or pSDIM1-EGFP. The immunoprecipitation procedure was as described previously [26] and precipitated complexes were boiled in protein loading buffer and separated by SDS-PAGE. The presence of SDIM1, DNAJB4, GST-SDIM1, and SDIM1-EGFP in the complex was revealed by Western blotting as described above.

### Yeast two-hybrid screening

Human cDNA encoding the full length SDIM1 protein was cloned into the pGBKT7 vector (Clontch, Palo Alto, CA, USA) to generate a chimaeric open reading frame encoding the *Gal*4 DNA binding domain and SDIM1 protein. This construct was introduced into *Saccharomyces cerevisiae *strain AH109. A single colony containing cells harboring the pGBKT7-SDIM1 plasmid was then used to provide host cells for screening a human brain cDNA expression library constructed using the pACT2 vector (Clontech, Palo Alto, CA, USA). The protein-protein interaction was first screened by plating the transformants onto SD/-Trp-Leu-His-Ade selection plates. Positive clones were then re-screened for the presence of β-galactosidase activity to eliminate false interactions. Library plasmids harboring SDIM1 interacting proteins were rescued and re-introduced into the pGBKT7/SDIM1-containing host cells to further eliminate false interactions. The identity of the cDNA encoding SDIM1-interacting protein was revealed by DNA sequencing and database searches.

## Competing interests

The authors declare that they have no competing interests.

## Authors' contributions

JXL performed most experiments and helped to analyze the data. CGC purified SDIM1-GST fusion protein and immumoaffinity purified anti-SDIM1 antibody. CL prepared mouse tissues and performed tissue Western analysis. QYL carried out the design of the study, analysis and interpretation of the data and preparation of the manuscript. All authors read and approved the final manuscript.
